# Does the Beck Cognitive Insight Scale Predict Response to Cognitive Remediation in Schizophrenia?

**DOI:** 10.1155/2016/6371856

**Published:** 2016-07-19

**Authors:** Audrey Benoit, Philippe-Olivier Harvey, Louis Bherer, Martin Lepage

**Affiliations:** ^1^Prevention and Early Intervention Program for Psychoses (PEPP-Montreal), Douglas Mental Health University Institute, 6875 LaSalle Boulevard, Montreal, QC, Canada H4H 1R3; ^2^Psychology Department, UQAM, C.P. 8888 Succursale Centre-ville, Montreal, QC, Canada H3C 3P8; ^3^Department of Psychiatry, McGill University, 1033 Pine Avenue West, Montreal, QC, Canada H3A 1A1; ^4^PERFORM, Concordia University, 7200 Sherbrooke Street West, Montreal, QC, Canada H4B 1R6; ^5^Centre de Recherche de l'Institut Universitaire de Gériatrie de Montréal, 4545 Queen Mary Street, Montreal, QC, Canada H3W 1W4

## Abstract

Cognitive remediation therapy (CRT) has emerged as a viable treatment option for people diagnosed with schizophrenia presenting disabling cognitive deficits. However, it is important to determine which variables can influence response to CRT in order to provide cost-effective treatment. This study's aim was to explore cognitive insight as a potential predictor of cognitive improvement after CRT. Twenty patients with schizophrenia completed a 24-session CRT program involving 18 hours of computer exercises and 6 hours of group discussion to encourage generalization of cognitive training to everyday activities. Pre- and posttest assessments included the CogState Research Battery and the Beck Cognitive Insight Scale (BCIS). Lower self-certainty on the BCIS at baseline was associated with greater improvement in speed of processing (*r*_*s*_ = −0.48; *p* < 0.05) and visual memory (*r*_*s*_ = −0.46; *p* < 0.05). The results of this study point out potential associations between self-certainty and cognitive improvement after CRT, a variable that can easily be measured in clinical settings to help evaluate which patients may benefit most from the intervention. They also underline the need to keep investigating the predictors of good CRT outcomes, which can vary widely between patients.

## 1. Introduction

Over the years, many types of psychosocial interventions for improving cognitive abilities have been developed, most of which are referred to as cognitive remediation therapy (CRT). Recent meta-analyses on the efficacy of CRT have shown significant improvement on a global composite score of cognition as well as in several distinct cognitive domains (attention/vigilance, speed of processing, verbal working memory, verbal learning and memory, reasoning/problem solving, and social cognition) [1–3]. Additionally, meta-analyses that have investigated symptoms and functioning using global composite scores tend to show small, yet significant improvements in symptom severity and moderate improvements in functioning following the CRT interventions [2, 3]. Although CRT has been shown to be effective, efforts should be made towards providing this type of intervention in the most cost-effective way for the psychiatric population. Further investigations are needed to determine which variations in treatment and patient characteristics yield the most significant improvements [3–5].

One potential patient characteristic influencing CRT efficacy may be the level of insight displayed by patients. Insight is a multifaceted construct that is often subdivided into clinical and cognitive insight. Clinical insight encompasses the awareness and attributions made towards illness, symptoms, and need for treatment [6] whereas cognitive insight can be construed as the metacognitive processes involved in reflecting on one's own experiences and thoughts (self-reflectiveness) and in the willingness to reevaluate beliefs (self-certainty) [7]. As a whole, poor insight, whether clinical or cognitive, is considered an obstacle for treatment delivery as well as community and psychosocial functioning [6]. It has also been associated with poorer cognitive performance [8]. When thinking of patient characteristics that may interfere with improvement in CRT, the metacognitive processes of cognitive insight are of special interest since they represent either a cognitive bias (self-certainty) or a cognitive style (self-reflectiveness) that is likely to influence the patients' experience of the intervention, beyond whether or not they are aware of their difficulties.

In the existing literature, cognitive insight as measured by the Beck Cognitive Insight Scale (BCIS) has shown interesting associations with verbal memory in first-episode psychosis patients. Specifically, greater self-certainty was significantly associated with poorer verbal memory [9] and later with smaller hippocampal volume [10], but no such correlations were found with clinical insight measures. Moreover, links have been found between the BCIS scores and the outcome of another type of psychosocial intervention: cognitive-behavioral therapy (CBT). For instance, improvements in BCIS scores were significantly correlated with improvements in positive and negative symptom levels [11, 12]. Studies have also found that greater BCIS scores from the outset of CBT predicted the decrease in the severity of positive and negative symptoms after treatment [11, 13]. Better cognitive insight may positively influence CBT outcome because the BCIS scores putatively reflect a willingness to revisit beliefs and to reappraise them (self-reflectiveness) as well as a level of flexibility conducive to change (self-certainty). If predispositions such as these can lead to being more receptive to CBT, it is possible they could have a similar positive influence on the outcome of other cognition-based psychosocial interventions, including CRT.

The objective of this study is to evaluate whether cognitive insight as measured with the BCIS is associated with cognitive improvement in patients suffering from enduring schizophrenia following CRT. We expect that patients with greater cognitive insight but with domain-specific cognitive impairment prior to starting CRT are more likely to improve on a standardized neurocognitive battery.

## 2. Methods

### 2.1. Participants

Treatment teams of a psychosis program within a public mental health institute referred patients with possible cognitive impairments. Clinicians were given a pamphlet containing information on cognition and the current study to encourage them to refer any patients they felt could benefit from CRT. This allowed us to receive as many referrals as possible while leaving it to our trained research assistants to determine patient eligibility. Patients not meeting eligibility criteria were contacted to explain why and were still offered to participate in the intervention if they wanted. If the patients decided to engage in the CRT, they were offered the same treatment as study patients but without a post-CRT evaluation, and their baseline data was not included in any subsequent analyses.

Patients were eligible if they (1) had received a diagnostic of a schizophrenia spectrum disorder from their psychiatrist, verifiable in their medical file, (2) were between 18 and 50 years of age, (3) showed sufficient clinical stability to sit through the 1-hour long CRT sessions, and (4) showed a deficit on at least one cognitive domain on an objective measure (1.5 standard deviations or less below average). Patients were excluded if (1) they had an IQ of 70 or less as determined by the WASI [14], (2) they met the criteria for an active substance or alcohol abuse diagnosis, (3) they had a traumatic brain injury within the past 3 years, or (4) they did not understand sufficiently the study or were not able to provide informed consent. Recruitment of patients was completed as part of a larger study on cognition and brain imaging for which the research protocol (including the study presented here) was approved by the appropriate research ethics boards.

### 2.2. Intervention

The CRT program was implemented in an intensive rehabilitation unit as part of a feasibility study destined to integrate CRT into standard practice. The group-based, computer-assisted CRT sessions were led by either one or two trained therapists from a team of four (one graduate student, one occupational therapist, one school teacher, and one clinical neuropsychologist). Groups were composed of two to six patients, on a rolling-admission basis. Each session started with 45 minutes of individual computer activities and ended with a 15-minute group discussion on cognition in everyday life and strategy coaching. This program was designed to include both the drill-and-practice and the strategy coaching approaches described in the literature [2, 3, 5, 15]. Computer activities were preselected to train speed of processing, attention, and memory during the first 12 sessions and memory and executive functions in the last 12 sessions. The software used included Math Arena*™* and Thinkin' Things*™* Collections 1, 2, and 3. These are commercially available software programs and feature engaging graphics, adjustable difficulty levels, and feedback.

### 2.3. Measurements

#### 2.3.1. Clinical Variables

Chart review provided a confirmation of the patient's diagnoses and medication information at the start and completion of CRT. Symptom levels were measured using the Scale for Assessment of Positive Symptoms (SAPS) [16] and the Scale for Assessment of Negative Symptoms (SANS) [17]. The SANS total score was calculated without the attention items because of their overlap with the cognitive variables. We also removed the items of poverty of content of speech (from the alogia scale) and inappropriate affect (from the affective flattening scale) since factor analytic studies showed these to belong to the disorganized symptom cluster [18, 19]. Symptoms were evaluated at baseline and after CRT completion for a better characterization of our sample.

#### 2.3.2. Cognitive Measures

Initial assessment of participants included the WASI [14] for IQ evaluation. Participants also completed the CogState Research Battery (CSRB) for the following domains: speed of processing, attention/vigilance, working memory, visual learning and memory, verbal learning and memory, reasoning/problem solving, and social cognition. Details on the nature of the tasks in the CSRB as well as their validity and reliability in schizophrenia have been described elsewhere [20, 21]. The WASI and CSRB were both administered at baseline; only the CSRB was readministered after the CRT.

#### 2.3.3. Insight Measure

The BCIS was administered before and after CRT [7]. Scores derived from the scale include Self-Reflectiveness and Self-Certainty. Self-reflectiveness is thought to be beneficial for cognitive insight; therefore, a high score on this scale suggests good cognitive insight. Self-certainty on the other hand is thought to be detrimental to cognitive insight and a high score on this scale suggests poor cognitive insight.

### 2.4. Statistical Analyses

A composite score was calculated for each cognitive domain in the CSRB by averaging the *z*-scores for all tests within each domain and a global composite score was calculated by averaging *z*-scores of all individual tests. *z*-scores were derived using normative data from 35 healthy subjects that were also recruited as part of the larger cognition and brain imaging study but did not take part in any treatment and were tested with the CSRB only once.  Table 1 shows the demographic variables on which they were matched to the patients. Pearson product-moment correlations were used initially to explore relationships between insight, cognitive performance, and change in cognitive performance. Since multiple correlations of *r* > 0.3 were found between baseline BCIS scores and baseline cognitive performance, partial correlations were subsequently used to evaluate the associations between baseline BCIS scores and change in cognitive performance while controlling for the baseline cognitive performance level.

All analyses were conducted using SPSS version 21 (SPSS, Chicago, IL, USA) and were two-tailed with a critical *p* value of 0.05, including for BCIS correlations due to the exploratory nature of correlating cognitive domains individually with the insight scores. In addition to *p*-values, percentage of variance explained was also considered (squared partial correlation coefficients). All variables were normally distributed.

## 3. Results

### 3.1. Demographic and Clinical Variables

Thirty-three patients were enrolled in the study and 20 completed the 24 sessions. Patients took on average 15.83 weeks to complete all 24 sessions (median: 15.64 weeks). Patients who did not complete all 24 sessions and that either could not be reached anymore or did not show up to follow-up appointments were considered to have dropped out of the study. Average sessions attended for those who dropped out were 5.54 (median: 3, range: 0–23). Only two patients completed most of the CRT (20 and 23 sessions) but could not be reached to schedule a follow-up appointment. Patients who dropped out of the study did not significantly differ from those who stayed on any of the demographic or clinical variables measured.  Table 2 shows the demographic and clinical variables for patients who completed the study, including follow-up measurements, when available, for completeness.

Among the clinical data, we have observed a statistically significant difference between the baseline SAPS total score and the follow-up SAPS total score (see Table 2). There were no other statistically significant differences between baseline and follow-up measurements available.

### 3.2. Baseline Insight and Change in Cognition

**Figure 1:**
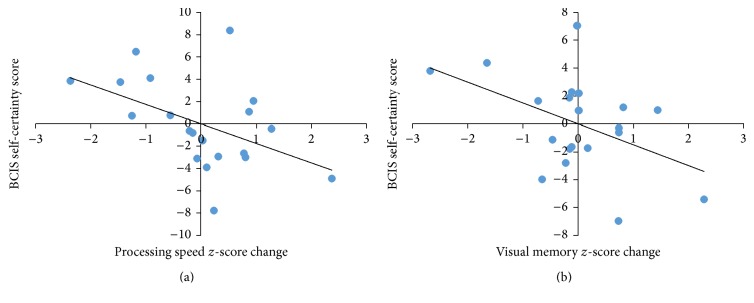
Partial correlations between baseline insight levels and improvement in cognition after CRT. Note: (a) baseline BCIS self-certainty score and improvement in speed of processing; (b) baseline BCIS self-certainty score and improvement in visual memory.

Table 3 shows the partial correlation coefficients for the three BCIS scores and each cognitive domain and Figure 1 shows the significant correlations. When baseline cognitive performance was controlled for, lower BCIS self-certainty at pretest was significantly associated with improvement in speed of processing (*p* = 0.039, variance explained: 23%) and visual memory (*p* = 0.045, variance explained: 22%). To verify that general intellectual ability did not play a role in cognitive change before and after CRT, the correlations between estimated full-scale IQ (WASI FSIQ) and change in cognitive performance in each domain were calculated. All correlations were found to be well over *p* = 0.100.

## 4. Discussion

The goal of this study was to investigate the relationship between patients' cognitive insight levels before starting a computer-assisted CRT and their subsequent improvement in cognition. After controlling for baseline cognitive performance, we found that higher initial cognitive insight, as measured with the self-certainty score only, was significantly correlated with greater improvement in speed of processing and visual memory. Hence, our study suggests that the BCIS whose self-administration is very brief, can be used to identify participants who are likely to benefit more from CRT. The differentiation we have found between self-reflectiveness and self-certainty is not surprising since the two variables are not extremes on a continuum, but rather different components of cognitive insight [7]. The constructs behind the BCIS scales capture the ability to objectively observe one's own mental productions (self-reflectiveness) and the resistance to correction (self-certainty). Ultimately, even if one can effectively reflect on his/her experiences and interpretations, if he/she is resistant to correction or overly certain about being right, this is likely to undermine the effectiveness of any psychosocial intervention, including CRT. When presented with the opportunity to try to improve their cognitive abilities, patients who believe their abilities are optimal, regardless of whether this is the case, may refuse the intervention. Notably, schizophrenia patients are thought to show limited insight into their cognitive impairments when they are present [23]. This type of insight is different from what is measured by the BCIS and can potentially explain why patients may or may not agree to participate in the intervention, and this was not our focus here. Once patients do agree to take part in CRT, we wanted to assess their metacognitive processes as measured by the BCIS as these can influence how patients experience CRT. While going through CRT, higher self-certainty about their own abilities may limit the patients' engagement in the computer activities or group participation. Although this is highly speculative, a predisposition to high self-certainty could even prevent motivation to shift from extrinsic to intrinsic, which is what our CRT program would hope to be able to accomplish. More precise investigations of these processes would be necessary to confirm these suggestions.

Questions also remain as to why only improvements in speed of processing and visual memory were linked to low self-certainty. In a recent meta-analysis including our previous work, Nair and colleagues [8] have observed that self-certainty inversely correlated with global cognitive performance, but not self-reflectiveness, and that at the domain level a significant inverse correlation was observed between self-certainty and memory. This supports a link between self-certainty and memory, but it remains unclear why we have found a significant correlation specifically with visual memory. This is especially true considering the results of our previous work, which points to an association of the BCIS measures with verbal memory rather than visual or working memory [9, 10]. However, this work was conducted in first-episode psychosis samples, which may limit its generalizability to the current study conducted with enduring schizophrenia patients. Considering that this is a first exploration of cognitive insight in relation to CRT outcome, the most likely explanation is that lower self-reflectiveness can have a general beneficial impact on CRT efficacy regardless of the cognitive domain, and that the domain-specific findings here are related to the study design. For instance, detectability of correlations could have been influenced by the different psychometric properties of cognitive tests on the CSRB, our small sample, or the modest improvement in performance following the intervention we have observed. Additional studies will be necessary to help elucidate these questions.

In addition to the aforementioned limitations, namely, the absence of a control group in the study design and small sample size, other limitations of this study include the absence of follow-up cognitive evaluation for patients who dropped out. Most patients had between 0 and 8 hours of CRT before dropping out, which we did not believe was enough to elicit significant changes in cognition but could have been verified. Additionally, we were not successful in scheduling the two patients who completed most of the therapy for a follow-up appointment. Consequently, our drop-out rate was high, close to 40%, which reduced our final sample significantly but was expected in a longitudinal study requiring approximately 28 visits over 3 to 4 months.

## 5. Conclusion

Although the statistical significance threshold was not corrected to preserve power in our small sample size, our findings point to a potential contributing factor to the heterogeneity observed in CRT outcomes. We believe these results warrant further examination, for example, in a randomized-controlled trial, to verify whether they can be replicated. Should the relationship highlighted here be confirmed, the BCIS could be a simple tool to help clinicians better anticipate CRT outcome. Ultimately, it would be interesting to investigate whether it is possible to lower self-certainty through psychosocial intervention and whether this would potentiate the effects of CRT on cognitive performance.

## Figures and Tables

**Table 1:** Demographic data of CogState research battery normative sample.

	Healthy controls (*n* = 35)	Patients (*n* = 20)	Statistic	*p*
Age (years)	32.89 (8.44)	35.55 (9.52)	*t* = −1.08	0.29
Gender (M : F)	21 : 14	13 : 7	*χ* ^2^ = 0.14	0.71
Parental socioeconomic status^a^	2.94 (0.84)	3.33 (0.84)	*χ* ^2^ = 2.93	0.23

^a^Measured by the Hollingshead two-factor index [22] in which 1 = highest and 4 = lowest.

**Table 2:** Demographic and clinical variables.

Patients who completed CRT (*n* = 20)	
	*M (SD)*
Years of education	11.65 (2.08)
Baseline antipsychotics total dose (mg/day in clz equivalents)	615.38 (539.89)
Follow-up antipsychotics total dose (mg/day in clz equivalents)	587.07 (414.45)
SAPS total, baseline^a^	25.26 (14.06)^b^
SAPS total, follow-up^a^	19.05 (13.05)^b^
SANS total, baseline^a^	26.42 (13.62)
SANS total, follow-up^a^	31.05 (12.95)
WASI FSIQ	90.30 (11.89)
Global cognitive composite score (CSRB), baseline	−1.51 (1.10)
Global cognitive composite score (CSRB), follow-up	−1.36 (1.17)
BCIS total score, baseline	2.70 (6.24)
BCIS total score, follow-up	2.75 (4.98)
*Diagnosis*	*n* (%)
Schizophrenia	1 (5)
Schizophrenia, paranoid	8 (40)
Schizoaffective disorder	6 (30)
Schizophrenia, undifferentiated	4 (20)
Psychosis NOS	1 (5)

^a^ *n* = 19; SAPS and SANS were not used for one patient.

^b^The difference in SAPS total score between baseline and follow-up is statistically significant (*t*_(18)_ = 2.41; *p* = 0.03).

**Table 3:** Partial correlations between change in cognitive performance and baseline insight levels.

Change in cognitive domain	Partial *r*	
	BCIS self-reflectiveness	BCIS self-certainty
Speed of processing	0.277	−0.476^*∗*^
Attention	−0.054	−0.083
Working memory	0.300	−0.075
Visual memory	0.313	−0.464^*∗*^
Verbal memory	0.229	−0.219
Executive functions	−0.141	−0.181
Social cognition	−0.198	−0.365
Global composite score	0.184	−0.103

^*∗*^ *p* < 0.05.

## References

[1] Grynszpan O., Perbal S., Pelissolo A. (2011). Efficacy and specificity of computer-assisted cognitive remediation in schizophrenia: a meta-analytical study. *Psychological Medicine*.

[2] McGurk S. R., Twamley E. W., Sitzer D. I., McHugo G. J., Mueser K. T. (2007). A meta-analysis of cognitive remediation in schizophrenia. *American Journal of Psychiatry*.

[3] Wykes T., Huddy V., Cellard C., McGurk S. R., Czobor P. (2011). A meta-analysis of cognitive remediation for schizophrenia: methodology and effect sizes. *American Journal of Psychiatry*.

[4] Vita A., Barlati S., Bellani M., Brambilla P. (2014). Cognitive remediation in schizophrenia: background, techniques, evidence of efficacy and perspectives. *Epidemiology and Psychiatric Sciences*.

[5] Barlati S., Deste G., De Peri L., Ariu C., Vita A. (2013). Cognitive remediation in schizophrenia: current status and future perspectives. *Schizophrenia Research and Treatment*.

[6] Lysaker P. H., Vohs J., Hillis J. D. (2013). Poor insight into schizophrenia: contributing factors, consequences and emerging treatment approaches. *Expert Review of Neurotherapeutics*.

[7] Beck A. T., Baruch E., Balter J. M., Steer R. A., Warman D. M. (2004). A new instrument for measuring insight: the Beck Cognitive Insight Scale. *Schizophrenia Research*.

[8] Nair A., Palmer E. C., Aleman A., David A. S. (2014). Relationship between cognition, clinical and cognitive insight in psychotic disorders: a review and meta-analysis. *Schizophrenia Research*.

[9] Lepage M., Buchy L., Bodnar M., Bertrand M.-C., Joober R., Malla A. (2008). Cognitive insight and verbal memory in first episode of psychosis. *European Psychiatry*.

[10] Buchy L., Czechowska Y., Chochol C. (2010). Toward a model of cognitive insight in first-episode psychosis: verbal memory and hippocampal structure. *Schizophrenia Bulletin*.

[11] Perivoliotis D., Grant P. M., Peters E. R., Ison R., Kuipers E., Beck A. T. (2010). Cognitive insight predicts favorable outcome in cognitive behavioral therapy for psychosis. *Psychosis*.

[12] Granholm E., Auslander L. A., Gottlieb J. D., McQuaid J. R., McClure F. S. (2006). Therapeutic factors contributing to change in cognitive-behavioral group therapy for older persons with schizophrenia. *Journal of Contemporary Psychotherapy*.

[13] Premkumar P., Peters E. R., Fannon D., Anilkumar A. P., Kuipers E., Kumari V. (2011). Coping styles predict responsiveness to cognitive behaviour therapy in psychosis. *Psychiatry Research*.

[14] Wechsler D. (1999). *Wechsler Abbreviated Scale of Intelligence*.

[15] Medalia A., Saperstein A. M. (2013). Does cognitive remediation for schizophrenia improve functional outcomes?. *Current Opinion in Psychiatry*.

[16] Andreasen N. C. (1984). *Scale for the Assessment of Positive Symptoms (SAPS)*.

[17] Andreasen N. C. (1984). *Scale for the Assessment of Negative Symptoms (SANS)*.

[18] Liddle P. F. (1987). The symptoms of chronic schizophrenia. A re-examination of the positive-negative dichotomy. *British Journal of Psychiatry*.

[19] Malla A. K., Norman R. M. G., Williamson P., Cortese L., Diaz F. (1993). Three syndrome concept of schizophrenia. A factor analytic study. *Schizophrenia Research*.

[20] Pietrzak R. H., Olver J., Norman T., Piskulic D., Maruff P., Snyder P. J. (2009). A comparison of the CogState Schizophrenia Battery and the Measurement and Treatment Research to Improve Cognition in Schizophrenia (MATRICS) Battery in assessing cognitive impairment in chronic schizophrenia. *Journal of Clinical and Experimental Neuropsychology*.

[21] Lees J., Applegate E., Emsley R. (2015). Calibration and cross-validation of MCCB and CogState in schizophrenia. *Psychopharmacology*.

[22] Hollingshead A. (1965). *Two-Factor Index of Social Position*.

[23] Medalia A., Thysen J. (2008). Insight into neurocognitive dysfunction in schizophrenia. *Schizophrenia Bulletin*.

